# Precise Electromagnetic Modulation of the Cell Cycle and Its Applications in Cancer Therapy

**DOI:** 10.3390/ijms26094445

**Published:** 2025-05-07

**Authors:** Keni Shi, Xiqing Peng, Ting Xu, Ziqi Lin, Mingyu Sun, Yiran Li, Qingyi Xian, Tingting Xiao, Siyuan Chen, Ying Xie, Ruihan Zhang, Jincheng Zeng, Bingzhe Xu

**Affiliations:** 1 School of Biomedical Engineering, Sun Yat-sen University, No. 135, Xingang Xi Road, Guangzhou 510275, China; shikn@mail2.sysu.edu.cn (K.S.);; 2 School of Biomedical Engineering, Shenzhen Campus of Sun Yat-sen University, No. 66, Gongchang Road, Guangming District, Shenzhen 518107, China; 3 Dongguan Key Laboratory of Medical Bioactive Molecular Developmental and Translational Research, Guangdong Medical University, Dongguan 523808, China; 4 Xinghai Institute of Cell, Dongguan 523808, China

**Keywords:** electromagnetic modulation, cell cycle control, cancer therapy, personalized treatment, tumor microenvironment

## Abstract

Precise modulation of the cell cycle via electromagnetic (EM) control presents a groundbreaking approach for cancer therapy, especially in the development of personalized treatment strategies. EM fields can precisely regulate key cellular homeostatic mechanisms such as proliferation, apoptosis, and repair by finely tuning parameters like frequency, intensity, and duration. This review summarizes the mechanisms through which EM fields influence cancer cell dynamics, highlighting recent developments in high-throughput electromagnetic modulation platforms that facilitate precise cell cycle regulation. Additionally, the integration of electromagnetic modulation with emerging technologies such as artificial intelligence, immunotherapy, and nanotechnology is explored, collectively enhancing targeting precision, immune activation, and therapeutic efficacy. A systematic analysis of existing clinical studies indicates that EM modulation technology significantly overcomes key challenges such as tumor heterogeneity, microenvironment complexity, and treatment-related adverse effects. This review summarizes the prospects of electromagnetic modulation in clinical translation and future research directions, emphasizing its critical potential as a core element in individualized and multimodal cancer treatment strategies.

## 1. Introduction

**Figure 1:**
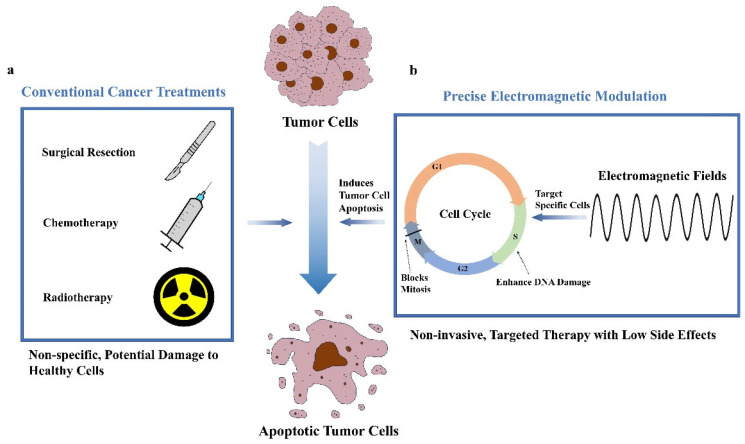
Comparison of conventional cancer treatments and precise electromagnetic modulation in cancer therapy. (**a**) Classification of conventional cancer treatments, including surgical resection, radiotherapy, and chemotherapy. (**b**) Schematic of the principle of precise electromagnetic modulation, which selectively modulates the tumor cell cycle and ultimately accelerates apoptosis. (G1:G1 phase First gap phase. G2:G2 phase Second gap phase. M:M phase Mitosis phase. S:S phase Synthesis phase.)

In recent years, the incidence and mortality rates of cancer have continued to rise globally, establishing it as a major public health concern and a leading cause of morbidity and mortality [1]. Conventional cancer treatments, including surgical resection, radiotherapy, and chemotherapy, though effective in controlling disease progression, possess considerable limitations. Surgical procedures often cannot fully excise all cancerous tissues and may cause significant harm to surrounding healthy tissues, affecting organ function [2]. Radiotherapy and chemotherapy, while crucial, are non-specific and may inadvertently damage healthy cells, leading to severe side effects and complications (Figure 1a) [3,4]. Therefore, there is an urgent need to develop alternative therapeutic strategies that are both targeted and minimally invasive.

The rapid proliferation of cancer cells is closely linked to dysregulation within the cell cycle. The cell cycle, which governs eukaryotic cell division and proliferation, is composed of several phases: G1, S, G2, and M phases [5]. A sophisticated network of regulatory mechanisms ensures orderly progression through these stages, maintaining cellular homeostasis [6]. However, cancer cells frequently evade this regulatory network, leading to uncontrolled cell proliferation, apoptosis resistance, and differentiation blockage [7]. Abnormal cell cycle control presents a promising therapeutic target, as interfering with key cell cycle phases can effectively induce cell cycle arrest or promote apoptosis in cancer cells.

Recently, precise electromagnetic (EM) modulation has garnered substantial attention in cancer therapy due to its unique benefits, including non-invasiveness, adaptability, and low side effect profiles [8]. This technology employs specific electromagnetic frequencies to modulate cell cycle progression, selectively inhibiting cancer cell proliferation and accelerating apoptosis (Figure 1b) [9]. Unlike conventional therapies, precise EM modulation does not rely on chemotherapeutic agents and allows for frequency adjustments tailored to specific cancer cell types, thereby reducing resistance and sparing normal cells from unnecessary damage. Studies suggest that electromagnetic fields can interfere with microtubule dynamics within cancer cells, blocking mitosis by arresting cells in the M phase, while also enhancing reactive oxygen species (ROS) production, which activates mitochondrial apoptosis pathways [10,11,12]. This non-invasive approach also reduces infection and scarring risks associated with surgical treatments, potentially shortening patient recovery times and lowering healthcare costs.

Although precise EM modulation shows significant promise in cancer treatment, current theoretical understanding and experimental data are insufficient for widespread clinical application [13]. The effects of different electromagnetic frequencies on various cancer cell types are still complex, and the therapeutic efficacy has yet to be thoroughly validated in clinical trials [14]. Further research is necessary to elucidate the precise mechanisms through which electromagnetic fields interact with the cell cycle in cancer cells and to establish optimal treatment protocols for diverse cancer types. This review aims to summarize recent advancements in cell cycle-targeted electromagnetic modulation, highlight its therapeutic potential in oncology, and provide insights into future research directions.

## 2. Cell Cycle and Tumor Growth

**Figure 2:**
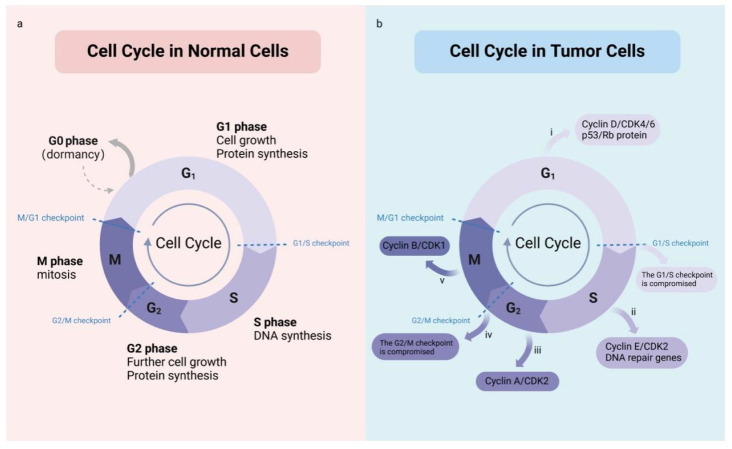
Overview of the cell cycle in normal and tumor cells. (**a**) The cell cycle typically consists of interphase (comprising G1, S, and G2 phases) and the mitotic (M) phase. (**b**) The dysregulation of cell cycle in tumor cells often involves aberrant expression and functional impairment of cell cycle regulatory molecules. (i) Overexpression or aberrant activation of Cyclin D and CDK4/6 results in rapid cell progress through the G1 phase to the S phase. Mutations or inactivation of p53 and Rb proteins make it impossible for cells to detect and repair DNA damage or prevent cells from entering the S phase. (ii) Overexpression or activation of Cyclin E and CDK2 promotes the progression of S phase. Mutations in DNA repair genes make it impossible for cells to repair DNA damage. (iii) Overexpression or activation of Cyclin A and CDK2 promotes rapid passage of cells through G2 phase to M phase. (iv) The inactivation of DNA damage checkpoint proteins prevents cells from effectively detecting and repairing DNA damage. (v) Overexpression or activation of Cyclin B and CDK1 promote cell progress.

The cell cycle encompasses a series of events through which a single cell divides to form two daughter cells. It typically consists of interphase (comprising G1, S, and G2 phases) and the mitotic (M) phase (Figure 2a) [15,16]. During the G1 phase, RNA and proteins essential for DNA replication are synthesized. In the S phase, chromosomal replication and DNA synthesis occur under precise regulatory control to ensure that each daughter cell receives a complete set of genetic information (Figure 2b). The G2 phase involves further growth, synthesis of additional RNA and proteins, and preparation for mitosis. The M phase marks the cessation of cellular growth and the physical division of the cell into two daughter cells, achieving an accurate distribution of genetic material between them. Each cell cycle stage is governed by a complex set of regulatory mechanisms, including various cell cycle checkpoints, which ensure that DNA replication and cell division proceed at the correct times and prevent damaged DNA from being transmitted to daughter cells [17].

In tumor cells, cell cycle regulation is disrupted, resulting in uncontrolled proliferation. This dysregulation often involves the aberrant expression and functional impairment of cell cycle regulatory molecules [18]. Cyclins, which are essential for advancing through specific cell cycle phases, are significantly upregulated in many cancers [19]. These proteins activate cell cycle transitions from one phase to the next. Meanwhile, Cyclin-dependent kinase inhibitors (CKIs) typically function to suppress Cyclin-dependent kinases (CDKs), halting cell cycle progression when necessary. However, in numerous cancers, CKIs lose their regulatory roles due to genetic mutations, downregulated expression, or functional inhibition, leading to uncontrolled cell cycle progression and excessive proliferation. In tumor cells, signaling pathways frequently upregulate positive regulators of the cell cycle, such as Cyclins and CDKs, or inhibit negative regulators, such as CKIs [20,21,22]. This enables tumor cells to bypass checkpoint restrictions and continuously progress through the cell cycle [23,24].

Unlike normal cells, tumor cells evade cell cycle regulation, resist natural clearance mechanisms, and achieve limitless proliferation [25]. Tumor-initiating cells, or cancer stem cells, are the foundation of this proliferative potential; they possess self-renewal and differentiation capabilities that sustain proliferative signals, evade growth suppression, and counteract apoptosis [26]. Tumor growth is further influenced by the tumor microenvironment, which provides nutritional and signaling support while suppressing normal immune responses, allowing sustained proliferation within the host body [27]. Dysregulated cell cycle control is fundamental to tumor development, and manipulating cell cycle checkpoints can enhance cancer cell sensitivity to chemotherapeutic agents, halting proliferation. Therefore, understanding cell cycle regulatory mechanisms, identifying inhibitors of key signaling pathways, and targeting specific cell cycle proteins offer viable strategies for cancer treatment [28]. CDK4 and CDK6, for instance, are major therapeutic targets, and several inhibitors, including palbociclib, ribociclib, and abemaciclib, have shown clinical efficacy against malignancies. Apart from CDK4/6, Cyclin E and CDK2 are potential targets, while inhibitors of checkpoint proteins such as ATM, ATR, CHK1, and WEE1 are under clinical investigation to increase tumor cell susceptibility to chemotherapy [29,30,31].

Targeted cell cycle therapies have demonstrated effectiveness for certain cancer types. However, the complexity and redundancy within the cell cycle regulatory network may limit the efficacy of highly selective CDK inhibitors. Although first-generation CDK inhibitors have broad activity across multiple CDKs and can disrupt the cell cycle and limit cell proliferation by reducing CDK enzyme activity, they have poor selectivity and high toxicity. Therefore, overcoming drug resistance in tumor cells and minimizing damage to normal cells are current research priorities in advancing cell cycle-targeted therapies. The development strategy for CDK4/6 inhibitors mainly focuses on the following six key areas: (1) Developing more specific inhibitors for CDK4/6 to reduce toxicity to normal cells [32]. (2) Exploring effective combination therapies to enhance efficacy and reduce resistance [33,34]. (3) Designing next-generation inhibitors that focus on improving chemical structures to overcome the limitations of traditional drugs and extend to targeting downstream molecules of CDK4/6, such as E2F transcription factors [35]. (4) Optimizing intermittent dosing regimens to alleviate side effects like inhibition [36]. (5) Identifying more biomarkers to provide a basis for patient selection [37]. (6) In-depth studies on resistance mechanisms have revealed causes of treatment failure, such as Rb mutations and CDK6 amplification, providing new targets to overcome resistance [38]. These strategies collectively drive the evolution of CDK4/6 inhibitors from first-generation to more effective and safer treatment options [39].

## 3. Electromagnetic Effects on Cells

Electromagnetic fields (EMFs) cover a broad spectrum of non-ionizing radiation, from static magnetic fields to visible light. There is no absolutely unified standard for classifying the frequencies in the field of bioelectromagnetics [40]. Depending on the research objectives, different researchers and institutions may use slightly different classification methods. The following introduces a commonly used classification: static fields (f = 0 Hz), extremely low frequency (ELF, 0 Hz < f ≤ 300 Hz), intermediate frequency (IF, 300 Hz < f ≤ 500 kHz), and radio frequency (RF, 500 kHz < f ≤ 300 GHz) [41,42]. Notably, most EMF frequencies utilized in therapeutic applications fall within the ELF range, highlighting their significance in treatment modalities. The biological effects of low-frequency magnetic fields are characterized by their non-invasive, non-ionizing, and non-thermal properties [41]. These fields can enhance cellular oxidative stress responses while also regulating apoptotic signaling pathways. Specifically, they can alter intracellular calcium (Ca^2+^) concentrations, leading to apoptosis [43,44]. This capacity to influence cellular signaling is central to the therapeutic efficacy of ELF-EMF exposure. In contrast, RF exposure is primarily associated with tissue heating, an effect that has been unequivocally demonstrated in various biological organisms [45]. Despite extensive research, no definitive deleterious effects, such as the development of cancer, have been observed in exposed animal subjects [25].

EMFs significantly influence cell cycle regulation through their interaction with intracellular metal ions and ion channels. Metal ions such as calcium, zinc, and iron serve as key cofactors in cellular processes, including signal transduction, energy metabolism, and DNA repair. EMFs can modulate the concentration and activity of these metal ions, thereby regulating cell proliferation and cell cycle progression. For instance, EMF affects intracellular calcium levels and distribution, which in turn regulates calcium-dependent signaling pathways, influencing various phases of the cell cycle, particularly the transition between G1 and S phases. Ca^2+^, as critical signaling molecules, directly regulate cell proliferation and division [46,47].

With the involvement of transition metals like iron and zinc, EMFs may alter the balance of metal ions within the cell, subsequently affecting the activity of enzymes related to the cell cycle. For example, Fe^2+^, by interacting with iron–sulfur clusters, regulates the function of several key enzymes, and EMFs can influence the activity of these enzymes by modulating iron states, thereby impacting the cell cycle [48]. Additionally, Fe^2+^ can interact with EMFs to form radical pairs, subsequently inducing effects such as DNA damage and lipid peroxidation. Zn^2+^ plays an important role in various stages of the cell cycle, and EMFs may also indirectly regulate key proteins and signaling pathways related to the cell cycle by altering zinc ion concentrations, thus affecting tumor cell proliferation [49].

EMFs also influence the activity of ion channels in the cell membrane, regulating cellular membrane potential and ion flow, which significantly impacts the cell cycle and proliferation. Ion channels such as calcium and sodium channels play a crucial role in cell cycle regulation, and EMFs can modulate these channels’ activities, adjusting intracellular concentrations of calcium, sodium, and potassium, and altering the cell’s membrane potential and physiological state [50]. This modulation of membrane potential and ion channel activity not only affects cell proliferation but may also promote or inhibit the entry of cells into apoptotic or autophagic pathways [51].

EMF exposure also induces oxidative stress by increasing free radical production and reducing antioxidant enzyme activity, potentially damaging biological macromolecules [52]. It elevates intracellular calcium levels by activating voltage-gated calcium channels (VGCCs), subsequently triggering protective responses through the nitric oxide–cGMP–protein kinase G signaling pathway [53]. Furthermore, EMF exposure has been shown to reduce cell viability by increasing membrane permeability. This effect is especially notable in studies examining breast cancer MCF-7 cells compared to normal MCF-10 cells, though similar impacts have been observed in human buccal epithelial cells and bacterial cells [25]. The influence of EMFs on cell proliferation and differentiation, particularly in stem cells, varies by frequency and intensity. Low frequencies tend to promote both proliferation and differentiation, especially in osteogenic and neuronal lineages [54]. Beyond these effects, EMF exposure can also lead to genotoxic outcomes, including increased DNA strand breaks and chromosomal abnormalities [55]. Finally, EMFs can induce cell death through both apoptosis and necrosis [56]. Evidence suggests that low-energy microwave radiation may specifically enhance apoptotic processes. However, it is important to note that cellular responses can vary significantly depending on exposure parameters and the quality of the studies conducted [57]. Electromagnetic fields (EMFs), particularly extremely low-frequency EMFs (ELF-EMFs), have drawn significant attention due to their complex interactions with living cells. Over the past 25 years, studies have explored how ELF-EMF exposure affects cellular and molecular behavior, with a focus on cancer cell metabolism and tumor progression [58]. This section reviews these effects, with emphasis on cellular electrophysiology and tumor cell cycle regulation. Exposure to ELF-EMF, especially at frequencies around 50/60 Hz, has been shown to alter the cell signaling pathways directly related to cell proliferation [52]. Such interference in signaling pathways affects various biological processes and potentially impacts cell growth and development. Studies suggest that ELF-EMF may disrupt redox signaling within cancer cells due to the distinctive electrical behaviors of these cells compared to normal cells [59]. Disruption of these critical signaling mechanisms can lead to programmed cell death (apoptosis) in cancer cells [60]. Thus, regulating signal transduction pathways through ELF exposure presents a promising approach for cancer therapy.

The tumor-suppressive effects of ELF-EMF have gained recent support, suggesting that ELF-EMF may exert inhibitory effects on tumors, notably in breast cancer. Pulsed low-frequency EMF (PMF) has emerged as an effective strategy to alter cancer cell membrane integrity, selectively damaging cancer cells without the need for ionizing radiation or cytotoxic drugs [61]. Consequently, PMF can serve as an adjunctive treatment to enhance the delivery of anticancer drugs to targeted tumor cells. These mechanisms underscore the potential of ELF-EMF in clinical settings by reducing the harmful side effects often associated with conventional cancer therapies. ELF-EMF also modulates the tumor microenvironment [62]. Trefoil Factor 2 (TFF2) is an anti-inflammatory peptide capable of inhibiting the proliferation of myeloid precursor cells. Research has shown that vagus nerve stimulation (VNS) can increase TFF2 expression, subsequently reducing the accumulation of myeloid-derived suppressor cells (MDSCs) in tumors [63]. This reduction is crucial for enhancing T cell immune responses, highlighting the role of the nervous system in tumor progression [64]. Therefore, electromagnetic stimulation may offer a therapeutic pathway to boost immune responses against tumors, minimizing the side effects associated with drug interventions.

Based on the current understanding of the interactions between electromagnetic fields (EMFs) and biological systems, as well as tumor cells, designing effective EMF-based cancer therapies requires several key factors to be considered. The optimization of frequency is the primary consideration in such treatments, as different types of cancer may respond best to specific frequency ranges [65]. At the same time, the precise control of field strength is equally crucial to ensure sufficient biological effects while avoiding potential harmful consequences [66]. Exposure duration and patterns are also important factors in treatment design, and the choice between intermittent or continuous exposure should be based on the significant impact of different exposure time patterns on cellular responses. Some cancers may respond better to pulsed EMF than to continuous EMF exposure [67].

Additionally, the design of EMF treatment should consider its synergistic effects with conventional therapies such as chemotherapy, radiotherapy, and immunotherapy to enhance overall efficacy [68]. The physical characteristics of the tumor microenvironment, such as tissue density, oxygenation status, and pH, can significantly influence EMF penetration and efficacy and thus should be incorporated into the treatment design [69].

Regarding biomarkers for predicting tumor sensitivity to EMFs, the expression level of voltage-gated calcium channels (VGCCs) is considered an important indicator. As the primary mediators of EMF effects, the differential expression of VGCC subtypes can predict tumor responsiveness. Not only L-type channels but also T-type, N-type, and P/Q-type channels may exhibit greater sensitivity to EMF exposure [70]. Intracellular redox markers, including reactive oxygen species (ROS) levels, antioxidant enzymes, and glutathione, can also serve as biomarkers for predicting EMF efficacy, as EMF is known to regulate oxidative stress responses [71]. Calcium-dependent signaling molecules, heat shock protein expression profiles (especially HSP70 and HSP90), as well as membrane potential and ion channel expression patterns may also serve as important biomarkers for predicting EMF treatment responses [72,73]. The reproducibility of EMF treatment across different types of tumors will benefit from the comprehensive characterization of these biomarkers in pre-treatment tumor biopsies, thus supporting the personalized selection of EMF parameters. Furthermore, the real-time monitoring of cellular responses during initial treatment can enable the adaptive optimization of EMF parameters for individual patients [74]. This field requires further systematic research to correlate these potential biomarkers with treatment outcomes across different cancer types, ultimately establishing validated predictive tools for EMF treatment efficacy.

Emerging evidence indicates that ELF-EMF affects the epigenetic mechanisms involved in cancer progression. This influence occurs through the regulation of DNA methylation, histone modifications, and microRNA (miRNA) expression, which are essential for maintaining cellular homeostasis [75,76]. These mechanisms disrupt fundamental processes like DNA damage repair, apoptosis, and cell cycle regulation. Notably, researchers found that a 0.2 T, 400 Hz non-sinusoidal magnetic field (NSMF) significantly suppressed Bcl-2 expression and upregulated caspases 3 and 9 in Bel-7402 cancer cells, indicating activation of the intrinsic apoptotic pathway and enhanced tumor cell death [77].

EMFs differ from pharmacological epigenome modifiers in multiple ways: EMFs primarily affect epigenetic states indirectly through oxidative stress [50], while epigenetic drugs directly target specific enzymes; EMFs lack specificity and affect multiple gene loci, whereas modern epigenetic drugs exhibit higher specificity [78]; EMFs demonstrate complex and inconsistent dose–effect relationships with nonlinear dose–response characteristics, while drugs show more predictable relationships [79]; the duration of EMF-induced epigenetic changes remains uncertain, whereas drug effects typically have well-defined characteristics [80]; and in terms of clinical applications, EMFs remain in the exploratory stage, while multiple epigenetic drugs have received FDA approval for cancer treatment [81].

## 4. The Effects of EMFs on Tumor Cell Dynamics and Synergistic Potential with Conventional Treatments

**Figure 3:**
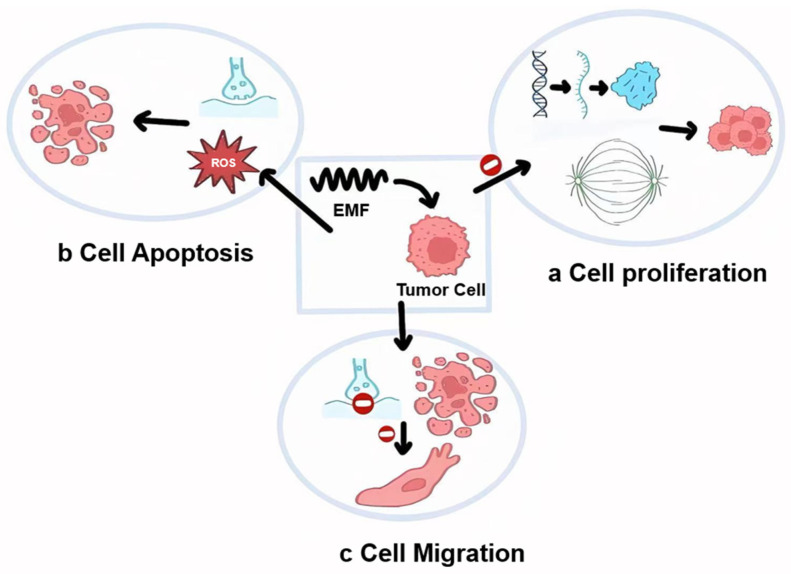
EMFs’ effect on tumor cells. (**a**) Inhibiting cell proliferation. (i) Altering gene expression. (ii) Disrupting mitotic spindles. (**b**) Promoting cell apoptosis. (i) Increasing ROS production. (ii) Regulating cellular signaling pathways. (**c**) Inhibiting cell migration. (i) Suppressing signaling pathways. (ii) Promoting tumor cell apoptosis.

Low-frequency EMFs exhibit a strong inhibitory effect on tumor cell proliferation while sparing normal cell growth. Studies reveal that extremely low-level 27.12 MHz radiofrequency EMFs, modulated at specific frequencies, can inhibit tumor cell growth by altering gene expression and disrupting mitotic spindles, with frequency-modulated anti-proliferative effects showing both tumor and tissue specificity. Additionally, low-intensity EMFs tend to be more effective in slowing down cell proliferation (Figure 3a) [82]. Exposure duration is also critical for the inhibition of cell proliferation. In tumor treatment, the optimization of electromagnetic field exposure parameters (frequency, intensity, and duration) directly affects clinical outcomes, but currently there is a lack of systematic guidelines for parameter selection. Electromagnetic field therapy has developed into various applications based on different tumor types, showing significant therapeutic potential. (1) Tumor-Treating Fields (TTFields) has been incorporated as a standard adjuvant treatment for glioblastoma multiforme (GBM), with an optimal frequency of 200–300 kHz determined by clinical trial data, requiring patients to use the device for at least 18 h daily to maintain anti-tumor effects [83]. (2) Radiofrequency ablation (RFA) is suitable for solid tumors such as liver, kidney, and lung cancers, operating at a frequency of 450–500 kHz with a treatment duration of 12–30 min, with these parameters being determined based on thermal effects and tissue penetration [84]. (3) Low-intensity pulsed ultrasound (LIPUS) is applied to tumors, with a frequency of 1–3 MHz and treatment sessions of 15–20 min, with parameter settings aimed at balancing tissue damage and therapeutic efficacy [85]. However, extended EMF exposure often enhances inhibitory effects but may negatively affect normal cells, thus requiring careful optimization. EMFs promote apoptosis in tumor cells by increasing reactive oxygen species (ROS) production and regulating cellular signaling pathways (Figure 3b) [86]. For example, ELF-EMFs have been found to induce apoptosis in human osteosarcoma cells by increasing ROS levels and activating the p38 MAPK pathway [87,88]. EMFs also activate mitochondrial pathways and extracellular signal-regulated kinase (ERK) pathways, contributing to apoptosis induction (Figure 3c) [89]. However, ELF-EMF-induced apoptosis varies depending on cell type; attached cancer and non-cancer cells show significant responses, whereas non-adherent cancer cells exhibit little reaction [90]. Studies indicate that low-frequency EMFs can promote migration in certain cancer cells [91]. On the other hand, certain frequencies and intensities of EMFs have been shown to inhibit tumor cell migration significantly by suppressing signaling pathways and promoting apoptosis [92,93]. EMFs also impact tumor cell morphology, membrane structure, metabolism, growth, adhesion, immune response, and microcirculation, contributing to reduced tumor recurrence risk [94].

EMFs exhibit notable synergy when combined with other treatments, such as physical therapies, radiotherapy, chemotherapy, and immunotherapy [95]. EMFs, when combined with other physical therapies, enhance tumor treatment outcomes. For instance, studies have shown that combining EMFs with ultrasound therapy can enhance local thermal effects, accelerating tumor cell apoptosis or necrosis [96]. Combining EMFs with radiotherapy significantly enhances the inhibitory effect on tumor cells, particularly by increasing tumor cell radiosensitivity [97,98]. Specific EMF frequencies not only increase tumor cell apoptosis but also inhibit DNA damage repair, thus boosting radiotherapy effectiveness [56]. EMFs demonstrate synergy with chemotherapy drugs, allowing for dose reduction while maintaining anticancer effects, which reduces side effects and increases patient tolerance. Research shows that EMFs can improve chemotherapy drug permeability, enhancing cytotoxicity against tumor cells [99]. For example, ELF-EMFs combined with paclitaxel increased the drug’s cytotoxic efficacy [43]. Additionally, alternating electric fields enhance chemotherapy by affecting cell membrane permeability, disrupting cancer cell division [100]. EMF application can enhance the efficacy of immunotherapy by increasing immune cell activity and enhancing penetration into the tumor microenvironment, thereby improving the anti-tumor response of immunotherapy [101,102]. Research indicates that EMF has synergistic effects with anti-PD-1/PD-L1 therapy, and EMF-induced ICD can enhance tumor antigen presentation and T cell activation. Furthermore, research indicates that the timing sequence between EMF and immunotherapy is crucial; EMF treatment prior to checkpoint inhibition may be more effective [103].

## 5. Precise Electromagnetic Regulation of the Cell Cycle

This section discusses the application of precise electromagnetic regulation on the cell cycle of tumor cells, which promises to bring revolutionary breakthroughs to cancer treatment. We will introduce the technology in three aspects of cell cycle regulation. Cellular electrophysiological signals, such as membrane potential [104,105], ion channel activity [106], and intracellular ion concentration, play a critical regulatory role at various stages of the cell cycle and are closely related to the health and functionality of the cell. A feedback control system based on electrophysiological signals can monitor changes in these signals and adjust the cellular environment or external stimuli accordingly, thereby maintaining or restoring the normal functional state of the cells. For example, during different cell cycle phases such as the G1 and G2 phases, variations in membrane potential and ionic concentrations can be dynamically tracked using technologies like embedded microelectrode arrays (RoMEA) [107], electrical impedance tomography (EIT) [108], and bioimpedance spectroscopy (BIS) [109]. Interventions can then be applied for precise targeting. By adjusting EMF frequency and intensity in real time based on feedback information, EMF can be targeted to affect tumor cells at specific stages while minimizing interference with normal cells.

**Figure 4:**
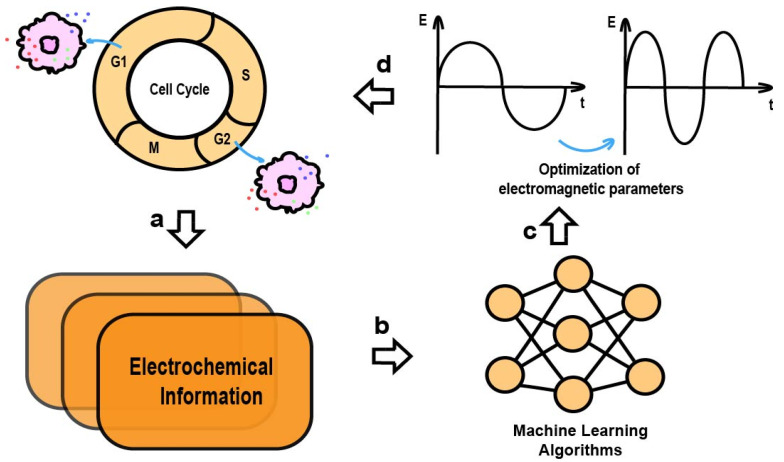
Feedback mechanisms based on electromagnetic modulation of cellular potentials. (**a**) Acquiring information about the potentials of different cell cycles. (**b**) Algorithm modeling based on acquired data. (**c**) Calculating the current optimal electromagnetic parameters according to the algorithm. (**d**) Applying the most appropriate parameters to the cell.

Different tumor cell types and their cycle dynamics respond differently to electromagnetic fields. Adaptive control methods [110] have been developed to track and suppress cancer cell proliferation and optimize electromagnetic device settings through multi-objective algorithms, which are central to the development of EMF control in cancer treatment. Through a feedback control system based on cellular electrophysiological signals, the precise targeting of electromagnetic regulation can be achieved. Constructing a feedback control system with sensors, signal processors, and actuators enables the real-time monitoring of target cells’ electrophysiological states, such as ion channel activity and membrane potential. This feedback system can adjust EMF parameters based on the cell’s cycle and physiological state, influencing activities like tumor cell cycle protein expression, apoptosis markers, and membrane permeability to achieve optimal inhibitory effects [111]. Machine learning and artificial intelligence algorithms are also helpful in designing electromagnetic parameters [112]. Machine learning algorithms have already been applied to analyze electrochemical signals [2]. For example, as illustrated in Figure 4, real-time monitoring of cell cycle-specific electrophysiological features, such as membrane potentials and ion concentrations, enables machine learning algorithms to analyze and learn from the data. This process facilitates the development of predictive models that determine how various tumor cell types respond to different EMF parameters, ultimately allowing for the selection of optimal parameters to regulate the tumor cell cycle. This facilitates the selection of optimal parameter combinations, improving accuracy and efficiency.

In recent years, several key breakthroughs in EMF regulation technology have promoted advancements in electromagnetic regulation therapy. Firstly, high-throughput screening (HTS) is a widely used experimental technique in biological and medical research. HTS, through automated processes and efficient data acquisition, can rapidly screen a large number of compounds or biological factors for specific bioactivities [113,114]. This technology, crucial in drug discovery, gene screening, and cell signaling studies [115,116,117], is also applicable to studying tumor cell electrophysiology under different EMF parameters. Advancements in electromagnetic field regulation equipment design are another critical area. By implementing specialized structural designs, these devices can provide a more stable EMF, enhancing precision and stability while reducing external interference [118,119]. They can also integrate real-time frequency monitoring and adaptive feedback mechanisms [120,121], allowing for automated frequency or intensity adjustments in line with different cell cycle phases. New gain and dissipation technologies enable devices to adjust EMF based on cellular feedback signals at various cycle stages, achieving the precise control of tumor cells. Together, high-throughput screening and the design of electromagnetic regulation equipment constitute the two primary innovations in EMF regulation technology. HTS can quickly evaluate the impact of numerous compounds or genetic variations on biological processes, facilitating the identification of sensitive cell populations for EMF-based modulation, thus providing a scientific foundation for precision therapy.

## 6. Application Cases and Challenges

**Figure 5:**
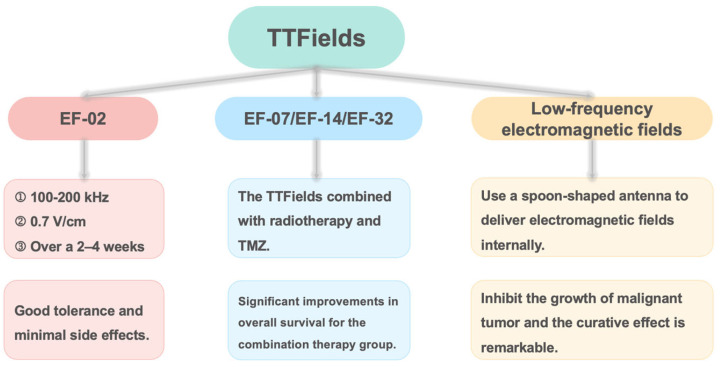
An introduction to the successful cases and efficacy evaluation of electromagnetic regulation in specific tumor treatment.

In recent years, electromagnetic modulation has made significant strides in cancer treatment (Figure 5), with various successful applications. One notable technique is Tumor-Treating Fields (TTFields), which has been extensively researched for treating different types of tumors, including glioblastoma, mesothelioma, and liver cancer [122,123]. TTFields utilizes specific frequencies and intensities of electric fields to disrupt the mitotic process of cancer cells, thereby inhibiting tumor growth and spread [124]. Its non-invasive nature and minimal impact on healthy cells present TTFields as a promising and relatively safe treatment option for cancer patients [125]. The first human trial using TTFields was the EF-02 study, which included patients with various tumor types such as breast cancer, malignant melanoma, pleural mesothelioma, and glioblastoma (GBM). Patients underwent TTFields therapy at frequencies of 100–200 kHz with an electric field strength of 0.7 V/cm over a 2–4-week period. Among six participants, one patient with metastatic breast cancer exhibited partial remission, and the treatment demonstrated good tolerability with an adherence rate of 80%, experiencing only grade 1 skin irritation as a side effect, showcasing the potential of TTFields [126,127].

In 2004, a pilot clinical trial (EF-07) on TTFields for treating GBM recruited 20 patients (10 with recurrent GBM and 10 newly diagnosed GBM) [128]. Newly diagnosed GBM patients received postoperative radiation therapy and adjuvant temozolomide (TMZ) chemotherapy, and received TTFields treatment while on maintenance TMZ therapy, and they showed progression-free survival (PFS) of 155 weeks and overall survival (OS) exceeding 39 months, significantly longer than contemporaneous control patients’ PFS and historical control patients’ OS [129]. Recurrent GBM patients received TTFields as the sole salvage therapy, having a median time to disease progression (TTP) of 26.1 weeks and a median OS of 62.2 weeks, which was more than double that of the historical controls. Ten years later, the EF-14 trial confirmed that the combination of TMZ with TTFields resulted in superior outcomes compared to TMZ alone, with a median PFS of 6.7 months and median OS of 20.9 months [130]. For patients experiencing their first recurrence, those receiving TTFields in combination with chemotherapy had a median survival of 11.8 months, compared to 9.2 months for those undergoing chemotherapy alone [131]. The prospective TIGER study (NCT03258021) involved 429 newly diagnosed glioblastoma patients treated with TTFields [132]. As of 1 June 2024, the results indicated a median overall survival of 19.6 months and a median progression-free survival of 10.2 months, with one-, two-, three-, and four-year survival rates of 79.2%, 42.4%, 31.5%, and 27.7%, respectively. This result was aligned with EF-14 and once again validates the effectiveness of TTFields in the real-world setting. The EF-32 trial further investigated the efficacy of the TTFields combined with radiotherapy and TMZ, showing significant improvements in overall survival for the combination therapy group [133].

In addition to glioblastoma, TTFields has shown significant therapeutic effects in a variety of other malignant tumors. Studies have demonstrated that TTFields significantly improves survival in patients with malignant pleural mesothelioma, with 1-year and 2-year survival rates of 62.2% and 41.9%, respectively, and no serious adverse events related to the treatment [134]. In non-small-cell lung cancer (NSCLC), TTFields effectively inhibits tumor cell proliferation and, when combined with chemotherapy drugs, significantly extends patient survival [135]. TTFields also shows good results in pancreatic cancer, significantly inhibiting cancer cell proliferation and improving patient survival [136]. For liver cancer patients, TTFields combined with sorafenib significantly enhances efficacy without causing systemic side effects [137]. Additionally, TTFields has shown synergistic effects in the treatment of ovarian cancer, gastric cancer, and other malignancies, particularly when used in combination with chemotherapy and immunotherapy, significantly improving patient survival rates [138,139]. These studies suggest that TTFields not only effectively treats glioblastoma but also shows great potential in the treatment of many other tumor types, offering a broad range of applications.

Despite the promising prospects of TTFields in certain clinical applications, this technology still faces multiple challenges. First, the significant structural differences in tissues across various organs of the human body necessitate the customization of electric field parameters and the optimization of treatment plans for specific tumor types, along with further clinical research [67]. Second, while TTFields has the advantage of being a non-invasive treatment, it can still cause discomfort such as skin irritation, redness, and pain, and the existing care protocols require improvement [140]. In certain cases, TTFields may also interfere with normal dividing cells, leading to adverse reactions. Additionally, patients may refuse to shave their heads due to esthetic concerns [141]. Moreover, the long-term effectiveness of TTFields and the potential issue of resistance deserve attention, as prolonged use may lead to adaptive changes in tumor cells, reducing treatment efficacy [142]. Finally, the high cost of the technology (approximately EUR 240,000) severely limits its widespread use in resource-limited areas [143]. Overcoming these limitations is crucial to fully unlock the therapeutic potential of TTFields and provide patients with safer and more effective cancer treatment options.

In addition to TTFields [144,145], high-frequency electromagnetic field therapy plays an important role in the treatment of solid tumors. High-frequency electromagnetic field therapy mainly includes radiofrequency ablation (RFA), microwave ablation (MWA), capacitive coupling high-frequency field hyperthermia, and electromagnetic nanoparticle hyperthermia. These techniques deliver precise electromagnetic energy to achieve the targeted treatment of tumor tissues while maximizing the protection of the surrounding healthy tissues. High-frequency electromagnetic field therapy has shown significant efficacy in a variety of solid tumors, with advantages such as minimal trauma, quick recovery, and repeatability, making it an important treatment option beyond surgery, radiotherapy, and chemotherapy.

Radiofrequency ablation (RFA) uses high-frequency electromagnetic fields around 500 kHz, delivered via an electrode needle inserted directly into tumor tissue, generating localized high temperatures (60–100 °C), which causes protein denaturation and irreversible cell death. In the treatment of liver cancer, long-term follow-up studies by Takayama and others have shown that for early-stage hepatocellular carcinoma patients with tumors ≤ 3 cm in diameter, the 5-year survival rate after RFA treatment can reach 40–60%, approaching the results of surgical resection, while significantly reducing complications and hospital stay time [146]. For inoperable lung cancer, a study by Simon et al. showed that the local tumor control rate after RFA treatment reached 78%, with 1-year, 2-year, and 3-year survival rates of 78%, 57%, and 36%, respectively, and about 90% of patients could be discharged within 24 h after RFA treatment [147]. In the treatment of bone metastases, RFA not only effectively alleviates pain but also reduces the risk of fractures and improves patients’ quality of life.

Microwave ablation (MWA) uses higher-frequency electromagnetic waves (915 MHz–2.45 GHz), which can generate a wider and more uniform thermal effect in a shorter amount of time, raising the tissue temperature to above 150 °C. Cui et al. analyzed and compared the effects of MWA and RFA in the treatment of hepatocellular carcinoma and found that for patients with moderately or poorly differentiated HCC, the 5-year overall survival rate in the MWA group was significantly higher, and the complete ablation rate was significantly higher than that of the RFA group [148].Liu et al. analyzed and suggested that radiofrequency ablation (RFA) appears to be more effective than microwave ablation (MWA) in the treatment of lung cancer/lung metastases (LC/LMs), showing better survival rates (overall survival, OS, and progression-free survival, PFS), with comparable safety. The advantage of PFS increases as survival time is prolonged [149].

Capacitive coupling high-frequency field hyperthermia (13.56 MHz) applies high-frequency electric fields to the target area through external electrode plates, maintaining tissue temperatures between 40 and 44 °C. It does not directly kill tumor cells but enhances their sensitivity to radiotherapy and chemotherapy and activates immune responses. A randomized clinical trial, EORTC 62961-ESHO 95, conducted by Issels et al. on 341 high-risk soft tissue sarcoma patients, showed that the median overall survival (67.3 months) in the local hyperthermia combined with chemotherapy group was significantly longer than the chemotherapy-only group (29.2 months) [150]. In the treatment of locally advanced breast cancer, a randomized multicenter study by the European Society for Hyperthermic Oncology (ESHO 1–85) demonstrated that adding hyperthermia as an adjunct to radiotherapy significantly improved treatment outcomes. This study found that the combination of hyperthermia and radiotherapy resulted in a higher local control rate and increased complete response rates compared to radiotherapy alone. This effect was particularly evident in patients with larger tumors or those who had failed previous radiotherapy. Additionally, the combined treatment was well tolerated, with no significant increase in side effects. This study supports the use of hyperthermia as an effective adjuvant therapy to enhance the efficacy of radiotherapy in locally advanced breast carcinoma [151].

Electromagnetic nanoparticle hyperthermia combines bio-targeted nanoparticles and high-frequency electromagnetic fields to achieve more precise tumor hyperthermia. This method first delivers magnetic nanoparticles (usually coated iron oxide particles) to the tumor tissue and then applies an alternating magnetic field (usually hundreds of kHz) to generate heat from the nanoparticles [152]. A study by Mahmoudi et al. showed that after combining magnetic nanoparticle hyperthermia with radiotherapy, the median survival was significantly longer than that for the historical control group, with mild treatment-related side effects [153].

Clinical applications have proven that these high-frequency electromagnetic field therapies have multiple advantages in tumor treatment: (1) minimal invasiveness, making them suitable for high-risk surgical patients; (2) precise targeting of the treatment area, reducing damage to normal tissues; (3) quick recovery, with most patients able to be discharged within 24–48 h; (4) repeatable application, especially suitable for recurrent or metastatic tumors; and (5) can be combined with other treatments (such as chemotherapy, radiotherapy, immunotherapy) to produce synergistic effects. With the advancement of imaging guidance technologies and improvements in electromagnetic field control accuracy, these treatment methods are rapidly evolving from palliative to curative therapies and are expected to play a more important role in future comprehensive cancer treatment.

A study conducted by Swiss, French, Brazilian, and American researchers highlighted remarkable results using low-frequency electromagnetic fields in treating malignant tumors. Patients used a spoon-shaped antenna to deliver electromagnetic fields internally, resulting in tumor volume reductions or stabilization after three weeks of treatment, while surrounding healthy cells remained unaffected. A clinical study on rotating magnetic field therapy enrolled 77 patients in the magnetic field (MF) group. Compared with the control group, more patients (66.7% vs. 25.9%) who received the low-frequency rotating magnetic field improved their quality of life at day 21 without an increase in adverse events [154].

**Figure 6:**
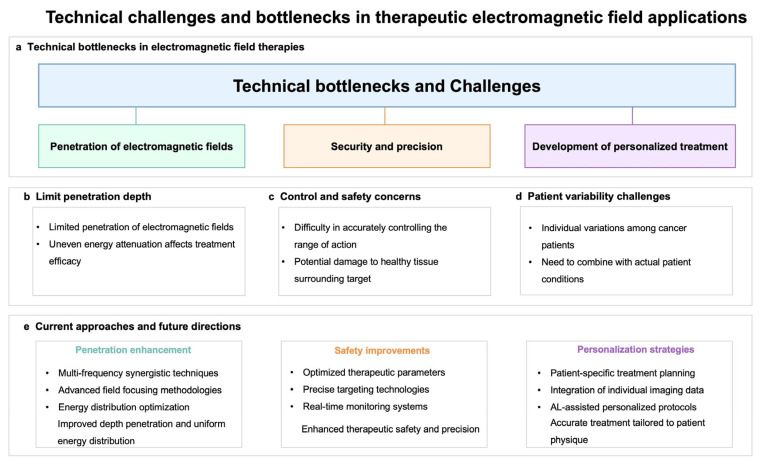
Overview of the current technical challenges of electromagnetic regulation in clinical transformation.

Despite the promise of electromagnetic modulation in clinical cancer treatment, several technical challenges remain, as shown in Figure 6. Firstly, the limited penetration of electromagnetic fields into biological tissues poses a significant challenge for treating deep-seated tumors [155]. Several methods can be employed to address the issue of electromagnetic field penetration depth in biological tissues. First, using higher-frequency electromagnetic fields can increase their penetration ability, but it is necessary to balance frequency with biological safety [156]. Secondly, combining magnetic nanoparticles (MNPs) can enhance the localized effect of the electromagnetic field under an external magnetic field, improving the treatment of deep-seated tumors [157]. Additionally, multimodal therapies, such as the combination of electromagnetic fields with hyperthermia, radiotherapy, or immunotherapy, can overcome the limitations of penetration depth [158]. Ultimately, adjusting the electric field strength and application method can also precisely target deep tumors while minimizing damage to normal tissues [159]. Integrating these methods can effectively improve the efficacy of electromagnetic field therapy for deep-seated tumors. Additionally, different tissue types exhibit varying absorption and scattering characteristics, leading to uneven energy attenuation during penetration, thereby affecting treatment efficacy. It is imperative to minimize damage to healthy tissues while employing electromagnetic fields for tumor treatment. However, the spatial resolution of electromagnetic fields is restricted by wavelength, making it difficult to precisely control the treatment area, potentially jeopardizing nearby healthy tissues [160]. Research on the effects of electromagnetic fields on normal cells and optimizing treatment parameters to enhance safety is essential. Due to individual variations among cancer patients, it is crucial to tailor electromagnetic modulation treatments to each patient’s specific circumstances [161]. Korshoej et al. systematically analyzed the impact of different electrode array positions on the field distribution in tumor regions and found that adjusting the electrode placement based on the tumor’s anatomical location significantly enhances the field strength in the tumor area, particularly in brain tumor treatment, where this adjustment is critical for optimizing the field distribution [162]. Ballo et al.’s research emphasizes the influence of tumor size on field strength distribution, noting that larger tumors require more precise electrode configuration to ensure adequate field coverage, thereby improving survival rates [142]. Mun et al. pointed out that different tumor types have varying sensitivities to electric field frequencies, such as NSCLC being most sensitive to 200 kHz, while ovarian cancer responds better to 150 kHz [83]. This difference may be related to the distinct cell membrane characteristics and intracellular structures of different tumors, suggesting that the optimal frequency should be adjusted based on the tumor type to enhance efficacy. Lacouture et al. studied the impact of patients’ skin conditions on the TTFields treatment and recommended adjusting field strength and treatment strategies based on patients’ skin sensitivity and previous treatment history to reduce adverse reactions [140]. Additionally, Kasey Rangan et al. pointed out that the special considerations for pediatric patients with gliomas necessitate the use of less harmful treatment methods [163]. In conclusion, personalized electromagnetic field treatment plans should comprehensively consider the tumor’s anatomical location, size, type, and the patient’s physiological characteristics.

Addressing these technical challenges is vital for unlocking the full potential of electromagnetic modulation as a viable treatment for various malignancies. While electromagnetic modulation presents great promise in cancer therapy, it is still regarded as an emerging treatment modality, facing numerous challenges that limit its acceptance in the broader medical community. The complex nature and variability of human tissues impact the penetration and effectiveness of electromagnetic fields, underscoring the need for ongoing research and innovation in this field.

## 7. Future Directions of Electromagnetic Control Technology in Tumor Treatment

**Figure 7:**
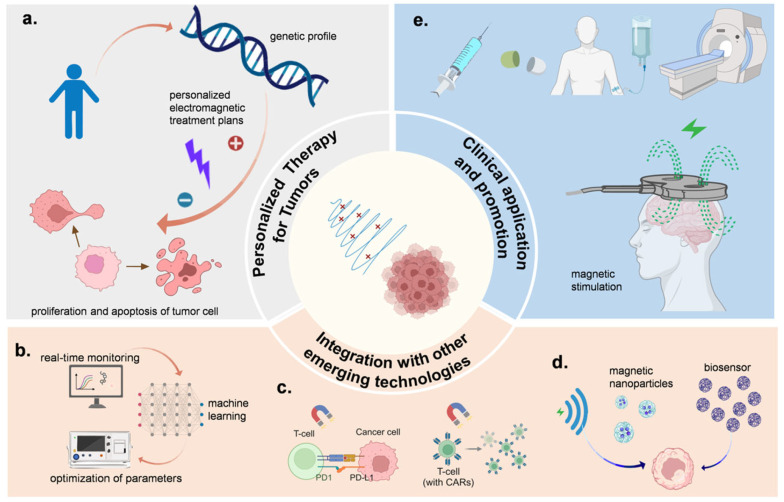
Future directions of electromagnetic control technology in tumor treatment. (**a**) Customized electromagnetic treatment plans tailored to each patient’s molecular and genetic profile. (**b**) Integration of artificial intelligence (AI) with electromagnetic therapy. (**c**) Combining electromagnetic modulation with immunotherapy. (**d**) Application of nanotechnology in electromagnetic modulation. (**e**) Electromagnetic modulation synergizes with traditional modalities.

Electromagnetic control technology holds significant promise in overcoming major challenges in cancer treatment, including tumor heterogeneity, the complexity of the tumor microenvironment, and therapeutic side effects [164,165]. This technology allows for highly specific spatiotemporal control, potentially enabling the modulation of tumor cell proliferation, apoptosis, and repair processes in a patient-specific manner. For instance, by targeting microtubule stability through magnetic field modulation, the metastatic potential of certain tumor cells can be reduced [166]. As illustrated in Figure 7a, customized electromagnetic treatment plans tailored to each patient’s molecular and genetic profile could improve treatment precision, with applications such as Tumor-Treating Fields (TTFields) therapy exemplifying the capacity to inhibit tumor cell growth without relying on tumor-specific mutations.

The integration of artificial intelligence (AI) with electromagnetic therapy has paved the way for increasingly personalized cancer treatments. AI algorithms can analyze vast clinical and electromagnetic parameter data [167,168], enabling real-time monitoring and optimization of electromagnetic field parameters based on cellular responses, as shown in Figure 7b. This smart system adapts electromagnetic exposure to enhance therapeutic efficacy [169]. Additionally, combining electromagnetic modulation with immunotherapy shows great potential, as electromagnetic fields can enhance tumor antigen presentation and stimulate immune responses [170]. Emerging applications include coupling with immune checkpoint inhibitors and CAR-T therapies to amplify anti-tumor immunity (Figure 7c) [171,172,173].

Nanotechnology has further improved electromagnetic targeting and therapeutic effectiveness [174]. Magnetic nanoparticles, for instance, can be designed to release drugs or heat in response to electromagnetic fields [175], thus allowing precision delivery to tumor sites (Figure 7d). These nanoparticles could also be used as biosensors to detect changes in the tumor microenvironment, integrating diagnostics with therapy [176]. However, the precise molecular mechanisms behind the cellular response to electromagnetic fields require further exploration, as does the optimal combination of electromagnetic fields with nanomaterials for clinical outcomes.

While still in the early stages, the clinical translation of electromagnetic control technology shows promise. Beyond the direct modulation of tumor cell activity, electromagnetic treatments can potentially synergize with traditional modalities like chemotherapy and radiotherapy [177,178], minimizing side effects and addressing tumor recurrence risks by activating immune cells in the tumor microenvironment or other means (Figure 7e). Future research should focus on understanding tumor-specific response mechanisms, establishing individualized electromagnetic control parameters, and developing multimodal treatment combinations [179]. Large-scale clinical trials are crucial for validating the safety and efficacy of these therapies [180,181], alongside refining the reliability and usability of electromagnetic devices for broader clinical application.

In conclusion, electromagnetic control technology represents a promising avenue for personalized cancer therapy, offering a novel approach that could be integrated into clinical oncology practices. Its ongoing development and clinical validation could provide not only alternative treatment options but also a foundation for future cancer research and innovative therapeutic strategies.

## List of Abbreviations

EM	Electromagnetic
G1 phase	First gap phase
S phase	Synthesis phase
G2 phase	Second gap phase
M phase	Mitosis phase
ROS	Reactive oxygen species
CKIs	Cyclin-Dependent Kinase Inhibitors
CDKs	Cyclin-Dependent Kinases
CDK4	Cyclin-Dependent Kinase 4
CDK6	Cyclin-Dependent Kinase 6
CDK2	Cyclin-Dependent Kinase 2
ATM	Ataxia Telangiectasia Mutated
ATR	Ataxia Telangiectasia and Rad3 Related
CHK1	Checkpoint Kinase 1
VGCCs	Voltage-gated calcium channels
EMF	Electromagnetic field
ELF	Extremely low-frequency
PMF	Pulsed low-frequency
TFF2	Trefoil Factor 2
VNS	Vagus nerve stimulation
MDSCs	Myeloid-derived suppressor cells
miRNA	microRNA
NSMF	Non-sinusoidal magnetic field
RoMEA	Relative oxygen metabolic efficiency assay
EIT	Electrical impedance tomography
BIS	Bioimpedance spectroscopy
HTS	High-throughput screening
GBM	Glioblastoma
TMZ	Temozolomide
PFS	Progression-free survival
OS	Overall survival
TTP	Time to progression
MF	Magnetic field
AI	Artificial intelligence
MNPs	Magnetic nanoparticles
RFA	Radiofrequency ablation
LIPUS	Low-intensity pulsed ultrasound
LIPUS	Low-intensity pulsed ultrasound
MWA	Microwave ablation

## References

[1] Siegel R.L., Giaquinto A.N., Jemal A. (2024). Cancer statistics, 2024. CA Cancer J. Clin..

[2] Trayanova N.A., Popescu D.M., Shade J.K. (2021). Machine Learning in Arrhythmia and Electrophysiology. Circ. Res..

[3] Garces A.H.I., Porta N., Graham T.A., Banerji U. (2023). Clinical trial designs for evaluating and exploiting cancer evolution. Cancer Treat. Rev..

[4] Kleinberg L., Sloan L., Grossman S., Lim M. (2019). Radiotherapy, Lymphopenia, and Host Immune Capacity in Glioblastoma: A Potentially Actionable Toxicity Associated With Reduced Efficacy of Radiotherapy. Neurosurgery.

[5] Pathania A.S., Chava H., Balusu R., Pasupulati A.K., Coulter D.W., Challagundla K.B. (2024). The crosstalk between non-coding RNAs and cell-cycle events: A new frontier in cancer therapy. Mol. Ther. Oncol..

[6] Diehl F.F., Sapp K.M., Heiden M.G.V. (2024). The bidirectional relationship between metabolism and cell cycle control. Trends Cell Biol..

[7] Matthews H.K., Bertoli C., de Bruin R.A.M. (2022). Cell cycle control in cancer. Nat. Rev. Mol. Cell Biol..

[8] Xu A., Wang Q., Lv X., Lin T. (2021). Progressive Study on the Non-thermal Effects of Magnetic Field Therapy in Oncology. Front. Oncol..

[9] Huang J., Wang L.Y., Wang B., Liu G., Wang W.C., Ge C.Q. (2024). Research Progress on Modulation of Electromagnetic Performance through Micro-nanostructure Design. J. Inorg. Mater..

[10] Choi Y.H. (2022). Tacrolimus Induces Apoptosis in Leukemia Jurkat Cells through Inactivation of the Reactive Oxygen Species-dependent Phosphoinositide-3-Kinase/Akt Signaling Pathway. Biotechnol. Bioprocess Eng..

[11] Wolf F.I., Torsello A., Tedesco B., Fasanella S., Boninsegna A., D’Ascenzo M., Grassi C., Azzena G.B., Cittadini A. (2005). 50-Hz extremely low frequency electromagnetic fields enhance cell proliferation and DNA damage: Possible involvement of a redox mechanism. Biochim. Biophys. Acta (BBA)-Mol. Cell Res..

[12] Pokorny J., Pokorny J., Vrba J. (2021). Generation of Electromagnetic Field by Microtubules. Int. J. Mol. Sci..

[13] Tuieng R.J., Cartmell S.H., Kirwan C.C., Sherratt M.J. (2021). The Effects of Ionising and Non-Ionising Electromagnetic Radiation on Extracellular Matrix Proteins. Cells.

[14] Berg H., GÜnther B., Hilger I., Radeva M., Traitcheva N., Wollweber L. (2010). Bioelectromagnetic Field Effects on Cancer Cells and Mice Tumors. Electromagn. Biol. Med..

[15] Wang Z. (2022). Cell Cycle Progression and Synchronization: An Overview. Methods in Molecular Biology.

[16] Gao S.W., Liu F. (2019). Novel insights into cell cycle regulation of cell fate determination. J. Zhejiang Univ.-Sci. B.

[17] Yam C.Q.X., Lim H.H., Surana U. (2022). DNA damage checkpoint execution and the rules of its disengagement. Front. Cell Dev. Biol..

[18] Jamasbi E., Hamelian M., Hossain M.A., Varmira K. (2022). The cell cycle, cancer development and therapy. Mol. Biol. Rep..

[19] Lim S., Kaldis P. (2013). Cdks, cyclins and CKIs: Roles beyond cell cycle regulation. Development.

[20] Cersosimo R.J. (2019). Cyclin-dependent kinase 4/6 inhibitors for the management of advanced or metastatic breast cancer in women. Am. J. Health-Syst. Pharm..

[21] Desnoyers A., Nadler M.B., Kumar V., Saleh R., Amir E. (2020). Comparison of treatment-related adverse events of different Cyclin-dependent kinase 4/6 inhibitors in metastatic breast cancer: A network meta-analysis. Cancer Treat. Rev..

[22] Xia P., Liu Y.N., Chen J.R., Cheng Z.K. (2019). Cell Cycle Proteins as Key Regulators of Postmitotic Cell Death. Yale J. Biol. Med..

[23] Liu J., Peng Y.H., Wei W.Y. (2022). Cell cycle on the crossroad of tumorigenesis and cancer therapy. Trends Cell Biol..

[24] Hamilton E., Infante J.R. (2016). Targeting CDK4/6 in patients with cancer. Cancer Treat. Rev..

[25] Shckorbatov Y.G. Impact of electromagnetic radiation on human and animal cells: Approaches, results, perspectives. Proceedings of the 2016 8th International Conference on Ultrawideband and Ultrashort Impulse Signals (UWBUSIS).

[26] Tian Z., Yu T., Liu J., Wang T., Higuchi A. (2023). Introduction to stem cells. Prog. Mol. Biol. Transl. Sci..

[27] Arneth B. (2020). Tumor Microenvironment. Medicina.

[28] Zou T., Lin Z. (2021). The Involvement of Ubiquitination Machinery in Cell Cycle Regulation and Cancer Progression. Int. J. Mol. Sci..

[29] Gupta N., Huang T.T., Horibata S., Lee J.M. (2022). Cell cycle checkpoints and beyond: Exploiting the ATR/CHK1/WEE1 pathway for the treatment of PARP inhibitor-resistant cancer. Pharmacol. Res..

[30] Chu C., Geng Y., Zhou Y., Sicinski P. (2021). Cyclin E in normal physiology and disease states. Trends Cell Biol..

[31] Liang H.Z., Zhu Y., Zhao Z.Y., Du J.T., Yang X.Y., Fang H., Hou X.B. (2022). Structure-Based Design of 2-Aminopurine Derivatives as CDK2 Inhibitors for Triple-Negative Breast Cancer. Front. Pharmacol..

[32] O’Leary B., Finn R.S., Turner N.C. (2016). Treating cancer with selective CDK4/6 inhibitors. Nat. Rev. Clin. Oncol..

[33] Spring L.M., Wander S.A., Andre F., Moy B., Turner N.C., Bardia A. (2020). Cyclin-dependent kinase 4 and 6 inhibitors for hormone receptor-positive breast cancer: Past, present, and future. Lancet.

[34] Klein M.E., Kovatcheva M., Davis L.E., Tap W.D., Koff A. (2018). CDK4/6 Inhibitors: The Mechanism of Action May Not Be as Simple as Once Thought. Cancer Cell.

[35] O’Brien N., Conklin D., Beckmann R., Luo T., Chau K., Thomas J., Mc Nulty A., Marchal C., Kalous O., von Euw E. (2018). Preclinical Activity of Abemaciclib Alone or in Combination with Antimitotic and Targeted Therapies in Breast Cancer. Mol. Cancer Ther..

[36] Patnaik A., Rosen L.S., Tolaney S.M., Tolcher A.W., Goldman J.W., Gandhi L., Papadopoulos K.P., Beeram M., Rasco D.W., Hilton J.F. (2016). Efficacy and Safety of Abemaciclib, an Inhibitor of CDK4 and CDK6, for Patients with Breast Cancer, Non-Small Cell Lung Cancer, and Other Solid Tumors. Cancer Discov..

[37] Chen W.J., Chang C.Y., Lin J.K. (2003). Induction of G1 phase arrest in MCF human breast cancer cells by pentagalloylglucose through the down-regulation of CDK4 and CDK2 activities and up-regulation of the CDK inhibitors p27(Kip) and p21(Cip). Biochem. Pharmacol..

[38] Gong X., Litchfield L.M., Webster Y., Chio L.C., Wong S.S., Stewart T.R., Dowless M., Dempsey J., Zeng Y., Torres R. (2017). Genomic Aberrations that Activate D-type Cyclins Are Associated with Enhanced Sensitivity to the CDK4 and CDK6 Inhibitor Abemaciclib. Cancer Cell.

[39] Song G.M., Liu J., Tang X., Zhong J., Zeng Y.H., Zhang X.D., Zhou J.B., Zhou J., Cao L., Zhang Q.F. (2024). Cell cycle checkpoint revolution: Targeted therapies in the fight against malignant tumors. Front. Pharmacol..

[40] Gholipour Hamedani B., Goliaei B., Shariatpanahi S.P., Nezamtaheri M. (2022). An overview of the biological effects of extremely low frequency electromagnetic fields combined with ionizing radiation. Prog. Biophys. Mol. Biol..

[41] Pophof B., Henschenmacher B., Kattnig D.R., Kuhne J., Vian A., Ziegelberger G. (2023). Biological Effects of Electric, Magnetic, and Electromagnetic Fields from 0 to 100 MHz on Fauna and Flora: Workshop Report. Health Phys..

[42] Levitt B.B., Lai H.C., Manville A.M. (2022). Effects of non-ionizing electromagnetic fields on flora and fauna, part 1. Rising ambient EMF levels in the environment. Rev. Environ. Health.

[43] Sun J., Tong Y., Jia Y., Jia X., Wang H., Chen Y., Wu J., Jin W., Ma Z., Cao K. (2023). Effects of extremely low frequency electromagnetic fields on the tumor cell inhibition and the possible mechanism. Sci. Rep..

[44] Ashdown C.P., Johns S.C., Aminov E., Unanian M., Connacher W., Friend J., Fuster M.M. (2020). Pulsed Low-Frequency Magnetic Fields Induce Tumor Membrane Disruption and Altered Cell Viability. Biophys. J..

[45] Cha D.I., Lee M.W., Jeong W.K., Ahn S.H., Kang T.W., Song K.D., Min J.H., Rhim H., Lim H.K. (2021). Rim-arterial enhancing primary hepatic tumors with other targetoid appearance show early recurrence after radiofrequency ablation. Eur. Radiol..

[46] Zhen C., Zhang G., Wang S., Wang J., Fang Y., Shang P. (2024). Electromagnetic fields regulate iron metabolism in living organisms: A review of effects and mechanism. Prog. Biophys. Mol. Biol..

[47] Schuermann D., Mevissen M. (2021). Manmade Electromagnetic Fields and Oxidative Stress-Biological Effects and Consequences for Health. Int. J. Mol. Sci..

[48] Kıvrak E.G., Yurt K.K., Kaplan A.A., Alkan I., Altun G. (2017). Effects of electromagnetic fields exposure on the antioxidant defense system. J. Microsc. Ultrastruct..

[49] Céspedes O., Ueno S. (2009). Effects of radio frequency magnetic fields on iron release from cage proteins. Bioelectromagnetics.

[50] Consales C., Merla C., Marino C., Benassi B. (2012). Electromagnetic fields, oxidative stress, and neurodegeneration. Int. J. Cell Biol..

[51] Funk R.H., Monsees T., Ozkucur N. (2009). Electromagnetic effects—From cell biology to medicine. Prog. Histochem. Cytochem..

[52] Falone S., Santini S., Cordone V., Di Emidio G., Tatone C., Cacchio M., Amicarelli F. (2018). Extremely Low-Frequency Magnetic Fields and Redox-Responsive Pathways Linked to Cancer Drug Resistance: Insights from Co-Exposure-Based In Vitro Studies. Front. Public Health.

[53] García-Minguillán O., Maestú C. (2021). 30 Hz, Could It Be Part of a Window Frequency for Cellular Response?. Int. J. Mol. Sci..

[54] Young A., Hunt T., Ericson M. (2021). The Slowest Shared Resonance: A Review of Electromagnetic Field Oscillations Between Central and Peripheral Nervous Systems. Front. Hum. Neurosci..

[55] Hack S.J., Kinsey L.J., Beane W.S. (2021). An Open Question: Is Non-Ionizing Radiation a Tool for Controlling Apoptosis-Induced Proliferation?. Int. J. Mol. Sci..

[56] Barati M., Darvishi B., Javidi M.A., Mohammadian A., Shariatpanahi S.P., Eisavand M.R., Madjid Ansari A. (2021). Cellular stress response to extremely low-frequency electromagnetic fields (ELF-EMF): An explanation for controversial effects of ELF-EMF on apoptosis. Cell Prolif..

[57] Lai H., Levitt B.B. (2024). Cellular and molecular effects of non-ionizing electromagnetic fields. Rev. Environ. Health.

[58] Parham F., Portier C.J., Chang X.Q., Mevissen M. (2016). The Use of signal-Transduction and Metabolic Pathways to Predict Human Disease Targets from Electric and Magnetic Fields Using *in vitro* Data in Human Cell Lines. Front. Public Health.

[59] Simkó M. (2007). Cell type specific redox status is responsible for diverse electromagnetic field effects. Curr. Med. Chem..

[60] Akbarnejad Z., Eskandary H., Dini L., Vergallo C., Nematollahi-Mahani S.N., Farsinejad A., Abadi M.F.S., Ahmadi M. (2017). Cytotoxicity of temozolomide on human glioblastoma cells is enhanced by the concomitant exposure to an extremely low-frequency electromagnetic field (100Hz, 100G). Biomed. Pharmacother..

[61] Mohamed A.F., Nasr M., Amer M.E., Abuamara T.M.M., Abd-Elhay W.M., Kaabo H.F., Matar E.E.R., El Moselhy L.E., Gomah T.A., Deban M.A.F. (2022). Anticancer and antibacterial potentials induced post short-term exposure to electromagnetic field and silver nanoparticles and related pathological and genetic alterations: In vitro study. Infect. Agents Cancer.

[62] Bergandi L., Lucia U., Grisolia G., Salaroglio I.C., Gesmundo I., Granata R., Borchiellini R., Ponzetto A., Silvagno F. (2022). Thermomagnetic Resonance Effect of the Extremely Low Frequency Electromagnetic Field on Three-Dimensional Cancer Models. Int. J. Mol. Sci..

[63] Wang L., Duan Y.F., Lu S.J., Sun J.F. (2023). Magnetic Nanomaterials Mediate Electromagnetic Stimulations of Nerves for Applications in Stem Cell and Cancer Treatments. J. Funct. Biomater..

[64] Erin N., Duymuş O., Oztürk S., Demir N. (2012). Activation of vagus nerve by semapimod alters substance P levels and decreases breast cancer metastasis. Regul. Pept..

[65] Buckner C.A., Buckner A.L., Koren S.A., Persinger M.A., Lafrenie R.M. (2015). Inhibition of cancer cell growth by exposure to a specific time-varying electromagnetic field involves T-type calcium channels. PLoS ONE.

[66] Markov M.S., Hazlewood C.F. (2009). Electromagnetic field dosimetry for clinical application. Environmentalist.

[67] Kirson E.D., Dbalý V., Tovarys F., Vymazal J., Soustiel J.F., Itzhaki A., Mordechovich D., Steinberg-Shapira S., Gurvich Z., Schneiderman R. (2007). Alternating electric fields arrest cell proliferation in animal tumor models and human brain tumors. Proc. Natl. Acad. Sci. USA.

[68] Mattsson M.O., Zeni O., Simkó M., Scarfì M.R. (2018). Editorial: Effects of Combined EMF Exposures and Co-exposures. Front. Public Health.

[69] Parate D., Kadir N.D., Celik C., Lee E.H., Hui J.H.P., Franco-Obregón A., Yang Z. (2020). Pulsed electromagnetic fields potentiate the paracrine function of mesenchymal stem cells for cartilage regeneration. Stem Cell Res. Ther..

[70] Pall M.L. (2015). Scientific evidence contradicts findings and assumptions of Canadian Safety Panel 6: Microwaves act through voltage-gated calcium channel activation to induce biological impacts at non-thermal levels, supporting a paradigm shift for microwave/lower frequency electromagnetic field action. Rev. Environ. Health.

[71] Yakymenko I., Tsybulin O., Sidorik E., Henshel D., Kyrylenko O., Kyrylenko S. (2016). Oxidative mechanisms of biological activity of low-intensity radiofrequency radiation. Electromagn. Biol. Med..

[72] Blank M., Goodman R. (2009). Electromagnetic fields stress living cells. Pathophysiology.

[73] Levin M. (2014). Molecular bioelectricity: How endogenous voltage potentials control cell behavior and instruct pattern regulation in vivo. Mol. Biol. Cell.

[74] Stupp R., Taillibert S., Kanner A.A., Kesari S., Steinberg D.M., Toms S.A., Taylor L.P., Lieberman F., Silvani A., Fink K.L. (2015). Maintenance Therapy With Tumor-Treating Fields Plus Temozolomide vs Temozolomide Alone for Glioblastoma: A Randomized Clinical Trial. JAMA.

[75] Leng Q.R., Ding J., Dai M.Y., Liu L., Fang Q., Wang D.W., Wu L.J., Wang Y. (2022). Insights Into Platelet-Derived MicroRNAs in Cardiovascular and Oncologic Diseases: Potential Predictor and Therapeutic Target. Front. Cardiovasc. Med..

[76] Shayeghan M., Forouzesh F., Ansari A.M., Javidi M.A. (2021). DNMT1 and miRNAs: Possible epigenetics footprints in electromagnetic fields utilization in oncology. Med. Oncol..

[77] Li J., Ma Y., Li N., Cao Y., Zhu Y. (2014). Natural static magnetic field-induced apoptosis in liver cancer cell. Electromagn. Biol. Med..

[78] Dai W., Qiao X., Fang Y., Guo R., Bai P., Liu S., Li T., Jiang Y., Wei S., Na Z. (2024). Epigenetics-targeted drugs: Current paradigms and future challenges. Signal Transduct. Target. Ther..

[79] Saliev T., Begimbetova D., Masoud A.-R., Matkarimov B. (2019). Biological effects of non-ionizing electromagnetic fields: Two sides of a coin. Prog. Biophys. Mol. Biol..

[80] Kelly A.D., Issa J.J. (2017). The promise of epigenetic therapy: Reprogramming the cancer epigenome. Curr. Opin. Genet. Dev..

[81] Jin N., George T.L., Otterson G.A., Verschraegen C., Wen H., Carbone D., Herman J., Bertino E.M., He K. (2021). Advances in epigenetic therapeutics with focus on solid tumors. Clin. Epigenet..

[82] Zimmerman J.W., Pennison M.J., Brezovich I., Yi N., Yang C.T., Ramaker R., Absher D., Myers R.M., Kuster N., Costa F.P. (2012). Cancer cell proliferation is inhibited by specific modulation frequencies. Br. J. Cancer.

[83] Mun E.J., Babiker H.M., Weinberg U., Kirson E.D., Von Hoff D.D. (2018). Tumor-Treating Fields: A Fourth Modality in Cancer Treatment. Clin. Cancer Res..

[84] Ahmed M., Solbiati L., Brace C.L., Breen D.J., Callstrom M.R., Charboneau J.W., Chen M.H., Choi B.I., de Baère T., Dodd G.D. (2014). Image-guided tumor ablation: Standardization of terminology and reporting criteria—A 10-year update. J. Vasc. Interv. Radiol..

[85] Wood A.K., Sehgal C.M. (2015). A review of low-intensity ultrasound for cancer therapy. Ultrasound Med. Biol..

[86] Jooyan N., Goliaei B., Bigdeli B., Faraji-Dana R., Zamani A., Entezami M., Mortazavi S.M.J. (2019). Direct and indirect effects of exposure to 900 MHz GSM radiofrequency electromagnetic fields on CHO cell line: Evidence of bystander effect by non-ionizing radiation. Environ. Res..

[87] Yuan L.Q., Wang C., Lu D.F., Zhao X.D., Tan L.H., Chen X. (2020). Induction of apoptosis and ferroptosis by a tumor suppressing magnetic field through ROS-mediated DNA damage. Aging.

[88] Amirinejad M., Eftekhar-Vaghefi S.H., Mahani S.N.N., Salari M., Yahyapour R., Ahmadi-Zeidabadi M. (2024). Exposure to Low-Frequency Radiation Changes the Expression of Nestin, VEGF, BCRP and Apoptosis Markers During Glioma Treatment Strategy: An *In Vitro* Study. Curr. Radiopharm..

[89] Ma T., Ding Q., Liu C., Wu H. (2023). Electromagnetic fields regulate calcium-mediated cell fate of stem cells: Osteogenesis, chondrogenesis and apoptosis. Stem Cell Res. Ther..

[90] Nezamtaheri M.S., Goliaei B., Shariatpanahi S.P., Ansari A.M. (2022). Differential biological responses of adherent and non-adherent (cancer and non-cancerous) cells to variable extremely low frequency magnetic fields. Sci. Rep..

[91] Zhang Y., Yan J., Xu H., Yang Y., Li W., Wu H., Liu C. (2018). Extremely low frequency electromagnetic fields promote mesenchymal stem cell migration by increasing intracellular Ca(2+) and activating the FAK/Rho GTPases signaling pathways in vitro. Stem Cell Res. Ther..

[92] Moori M., Norouzian D., Yaghmaei P., Farahmand L. (2024). Electromagnetic field as a possible inhibitor of tumor invasion by declining E-cadherin/N-cadherin switching in triple negative breast cancer. Electromagn. Biol. Med..

[93] Tanzhu G., Chen L., Xiao G., Shi W., Peng H., Chen D., Zhou R. (2022). The schemes, mechanisms and molecular pathway changes of Tumor Treating Fields (TTFields) alone or in combination with radiotherapy and chemotherapy. Cell Death Discov..

[94] Zhang G., Liu X., Liu Y., Zhang S., Yu T., Chai X., He J., Yin D.-C., Zhang C.-Y. (2023). The effect of magnetic fields on tumor occurrence and progression: Recent advances. Prog. Biophys. Mol. Biol..

[95] Alshahat M.A., Elgenedy M.A., Aboushady A.A., Williams M.T.S. (2023). Cancer Treatment: An Overview of Pulsed Electric Field Utilization and Generation. Appl. Sci..

[96] Hosseinpour A., Soltani M., Souri M. (2024). Improving tumor treatment through intratumoral injection of drug-loaded magnetic nanoparticles and low-intensity ultrasound. Sci. Rep..

[97] Obrador E., Jihad-Jebbar A., Salvador-Palmer R., López-Blanch R., Oriol-Caballo M., Moreno-Murciano M.P., Navarro E.A., Cibrian R., Estrela J.M. (2023). Externally Applied Electromagnetic Fields and Hyperthermia Irreversibly Damage Cancer Cells. Cancers.

[98] Salinas-Asensio M.M., Ríos-Arrabal S., Artacho-Cordón F., Olivares-Urbano M.A., Calvente I., León J., Núñez M.I. (2019). Exploring the radiosensitizing potential of magnetotherapy: A pilot study in breast cancer cells. Int. J. Radiat. Biol..

[99] Yadegari Dehkordi S., Firoozabadi S.M., Forouzandeh Moghadam M., Shankayi Z. (2021). Endocytosis induction by high-pulsed magnetic fields to overcome cell membrane barrier and improve chemotherapy efficiency. Electromagn. Biol. Med..

[100] Homami E., Goliaei B., Shariatpanahi S.P., Habibi-Kelishomi Z. (2023). Alternating electric fields can improve chemotherapy treatment efficacy in blood cancer cell U937 (non-adherent cells). BMC Cancer.

[101] Tota M., Jonderko L., Witek J., Novickij V., Kulbacka J. (2024). Cellular and Molecular Effects of Magnetic Fields. Int. J. Mol. Sci..

[102] Fuster M.M. (2024). Integrating electromagnetic cancer stress with immunotherapy: A therapeutic paradigm. Front. Oncol..

[103] Messenheimer D.J., Jensen S.M., Afentoulis M.E., Wegmann K.W., Feng Z., Friedman D.J., Gough M.J., Urba W.J., Fox B.A. (2017). Timing of PD-1 Blockade Is Critical to Effective Combination Immunotherapy with Anti-OX40. Clin. Cancer Res..

[104] Lucia U., Grisolia G. (2020). Seebeck-Peltier Transition Approach to Oncogenesis. Appl. Sci..

[105] Gottschalk B., Koshenov Z., Malli R., Graier W.F. (2024). Implications of mitochondrial membrane potential gradients on signaling and ATP production analyzed by correlative multi-parameter microscopy. Sci. Rep..

[106] Maliszewska-Olejniczak K., Bednarczyk P. (2024). Novel insights into the role of ion channels in cellular DNA damage response. Mutat. Res.-Rev. Mutat. Res..

[107] Yang T.R., Huang D., Li C.H., Zhao D.Y., Li J.S., Zhang M.J., Chen Y.F., Wang Q.N., Liang Z.C., Liang X.J. (2021). Rolling microneedle electrode array (RoMEA) empowered nucleic acid delivery and cancer immunotherapy. Nano Today.

[108] Ihsan M.F., Kawashima D., Li S.S., Ogasawara S., Murata T., Takei M. (2024). Non-invasive hERG channel screening based on electrical impedance tomography and extracellular voltage activation (EIT-EVA). Lab A Chip.

[109] Oh T.I., Kang M.J., Jeong Y.J., Zhang T., Yeo S.G., Park D.C. (2021). Tissue Characterization Using an Electrical Bioimpedance Spectroscopy-Based Multi-Electrode Probe to Screen for Cervical Intraepithelial Neoplasia. Diagnostics.

[110] Ramaswamy V.D., Keidar M. (2024). Personalized Plasma Medicine for Cancer: Transforming Treatment Strategies with Mathematical Modeling and Machine Learning Approaches. Appl. Sci..

[111] Hernández-Bule M.L., Medel E., Colastra C., Roldán R., Ubeda A. (2019). Response of neuroblastoma cells to RF currents as a function of the signal frequency. BMC Cancer.

[112] Besler E., Wang Y.C., Sahakian A.V. (2020). Early and Late Fusion Machine Learning on Multi-Frequency Electrical Impedance Data to Improve Radiofrequency Ablation Monitoring. IEEE J. Biomed. Health Inform..

[113] Grubb L.M., Caliari R.S. (2021). Fabrication approaches for high-throughput and biomimetic disease modeling. Acta Biomater..

[114] Zuo J., Fang Y., Wang R., Liang S.S. (2024). High-throughput solutions in tumor organoids: From culture to drug screening. Stem Cells.

[115] Yamaguchi K., Nagatoishi S., Tsumoto K., Furukawa Y. (2020). Discovery of chemical probes that suppress Wnt/β-catenin signaling through high-throughput screening. Cancer Sci..

[116] Vercauteren S., Fiesack S., Maroc L., Verstraeten N., Dewachter L., Michiels J., Vonesch S.C. (2024). The rise and future of CRISPR-based approaches for high-throughput genomics. FEMS Microbiol. Rev..

[117] Huang Y., Shang M.Q., Liu T.T., Wang K.J. (2022). High-throughput methods for genome editing: The more the better. Plant Physiol..

[118] Zhang Y.F., Lu M. (2024). Advances in magnetic induction hyperthermia. Front. Bioeng. Biotechnol..

[119] Singh S., Repaka R. (2018). Parametric sensitivity analysis of critical factors affecting the thermal damage during RFA of breast tumor. Int. J. Therm. Sci..

[120] Baik J., Lee S., Yang S., Park S.M. (2022). Modularized Electrosurgical System With a Hybrid CPU-FPGA Chip for Real-Time Thermal Lesion Approximation. IEEE Trans. Instrum. Meas..

[121] Chai Z.P., Lyu L.X., Pu M.H., Chen X.W., Zhu J.Q., Liang H.G., Ding H., Wu Z.G. (2022). An Individually Controlled Multitined Expandable Electrode Using Active Cannula-Based Shape Morphing for On-Demand Conformal Radiofrequency Ablation Lesions. Adv. Intell. Syst..

[122] Yu A., Zeng J., Yu J.H., Cao S., Li A.L. (2024). Theory and application of TTFields in newly diagnosed glioblastoma. Cns Neurosci. Ther..

[123] Grosu A., Touchefeu Y., Brunner T., Gkika E., Thimme R., Cubillo A. (2020). phase 2 HEPANOVA study of tumor treating fields (TTFields, 150 kHz) concomitant with sorafenib in advanced hepatocellular carcinoma (HCC): Interim safety analysis. Ann. Oncol..

[124] Moser J.C., Salvador E., Deniz K., Swanson K., Tuszynski J., Carlson K.W., Karanam N.K., Patel C.B., Story M., Lou E.M. (2022). The Mechanisms of Action of Tumor Treating Fields. Cancer Res..

[125] Rominiyi O., Vanderlinden A., Clenton S.J., Bridgewater C., Al-Tamimi Y., Collis S.J. (2021). Tumour treating fields therapy for glioblastoma: Current advances and future directions. Br. J. Cancer.

[126] Salzberg M., Kirson E., Palti Y., Rochlitz C. (2008). A Pilot Study with Very Low-Intensity, Intermediate-Frequency Electric Fields in Patients with Locally Advanced and/or Metastatic Solid Tumors. Onkologie.

[127] Guo X., Yang X., Wu J., Yang H., Li Y., Li J., Liu Q., Wu C., Xing H., Liu P. (2022). Tumor-Treating Fields in Glioblastomas: Past, Present, and Future. Cancers.

[128] Kirson E.D., Gurvich Z., Schneiderman R., Dekel E., Itzhaki A., Wasserman Y., Schatzberger R., Palti Y. (2004). Disruption of cancer cell replication by alternating electric fields. Cancer Res..

[129] Kirson E.D., Schneiderman R.S., Dbaly V., Tovarys F., Vymazal J., Itzhaki A., Mordechovich D., Gurvich Z., Shmueli E., Goldsher D. (2009). Chemotherapeutic treatment efficacy and sensitivity are increased by adjuvant alternating electric fields (TTFields). BMC Med. Phys..

[130] Stupp R., Taillibert S., Kanner A., Read W., Steinberg D.M., Lhermitte B., Toms S., Idbaih A., Ahluwalia M.S., Fink K. (2017). Effect of Tumor-Treating Fields Plus Maintenance Temozolomide vs Maintenance Temozolomide Alone on Survival in Patients With Glioblastoma A Randomized Clinical Trial. JAMA-J. Am. Med. Assoc..

[131] Kesari S., Ram Z., Investigators E.F.T. (2017). Tumor-treating fields plus chemotherapy versus chemotherapy alone for glioblastoma at first recurrence: A post hoc analysis of the EF-14 trial. CNS Oncol..

[132] Bähr O., Tabatabai G., Fietkau R., Goldbrunner R., Glas M. (2024). Tumor treating fields (TTFields) therapy in patients with glioblastoma: Long-term survival results from TTFields in Germany in routine clinical care (TIGER) study. J. Clin. Oncol..

[133] Metsemakers W.J., Smeets B., Nijs S., Hoekstra H. (2017). Infection after fracture fixation of the tibia: Analysis of healthcare utilization and related costs. Inj.-Int. J. Care Inj..

[134] Ceresoli G.L., Aerts J.G., Dziadziuszko R., Ramlau R., Cedres S., van Meerbeeck J.P., Mencoboni M., Planchard D., Chella A., Crinò L. (2019). Tumour Treating Fields in combination with pemetrexed and cisplatin or carboplatin as first-line treatment for unresectable malignant pleural mesothelioma (STELLAR): A multicentre, single-arm phase 2 trial. Lancet Oncol..

[135] Tian W., Ning J., Chen L., Zeng Y., Shi Y., Xiao G., He S., Tanzhu G., Zhou R. (2024). Cost-effectiveness of tumor-treating fields plus standard therapy for advanced non-small cell lung cancer progressed after platinum-based therapy in the United States. Front. Pharmacol..

[136] Rivera F., Benavides M., Gallego J., Guillen-Ponce C., Lopez-Martin J., Küng M. (2019). Tumor treating fields in combination with gemcitabine or gemcitabine plus nab-paclitaxel in pancreatic cancer: Results of the PANOVA phase 2 study. Pancreatology.

[137] Davidi S., Jacobovitch S., Shteingauz A., Martinez-Conde A., Braten O., Tempel-Brami C., Zeevi E., Frechtel-Gerzi R., Ene H., Dor-On E. (2022). Tumor Treating Fields (TTFields) Concomitant with Sorafenib Inhibit Hepatocellular Carcinoma In Vitro and In Vivo. Cancers.

[138] Neuhaus E., Zirjacks L., Ganser K., Klumpp L., Schüler U., Zips D., Eckert F., Huber S.M. (2019). Alternating Electric Fields (TTFields) Activate Cav1.2 Channels in Human Glioblastoma Cells. Cancers.

[139] Chen A.B., Kotecha R., Chaney M., Balestrieri K., Gabrail N., Langer C. (2024). The Keynote B36 (EF-36) Pilot Study of Tumor Treating Fields (TTFields) Therapy with pembrolizumab as First-Line Treatment of PD-L1-Positive, Advanced or Metastatic Non-Small Cell Lung Cancer. Int. J. Radiat. Oncol. Biol. Phys..

[140] Lacouture M.E., Anadkat M.J., Ballo M.T., Iwamoto F., Jeyapalan S.A., La Rocca R.V., Schwartz M., Serventi J.N., Glas M. (2020). Prevention and Management of Dermatologic Adverse Events Associated With Tumor Treating Fields in Patients With Glioblastoma. Front. Oncol..

[141] Karanam N.K., Srinivasan K., Ding L., Sishc B., Saha D., Story M.D. (2017). Tumor-treating fields elicit a conditional vulnerability to ionizing radiation via the downregulation of BRCA1 signaling and reduced DNA double-strand break repair capacity in non-small cell lung cancer cell lines. Cell Death Dis..

[142] Ballo M.T., Urman N., Lavy-Shahaf G., Grewal J., Bomzon Z., Toms S. (2019). Correlation of Tumor Treating Fields Dosimetry to Survival Outcomes in Newly Diagnosed Glioblastoma: A Large-Scale Numerical Simulation-Based Analysis of Data from the Phase 3 EF-14 Randomized Trial. Int. J. Radiat. Oncol. Biol. Phys..

[143] Mittal S., Klinger N.V., Michelhaugh S.K., Barger G.R., Pannullo S.C., Juhász C. (2018). Alternating electric tumor treating fields for treatment of glioblastoma: Rationale, preclinical, and clinical studies. J. Neurosurg..

[144] Cao X., Li J., Ren J., Peng J., Zhong R., He J., Xu T., Yu Z., Jin H., Hao S. (2024). Minimally-invasive implantable device enhances brain cancer suppression. EMBO Mol. Med..

[145] Yang Y., Hu X., Liu Y., Ouyang B., Zhang J., Jin H., Yu Z., Liu R., Li Z., Jiang L. (2022). An implantable ultrasound-powered device for the treatment of brain cancer using electromagnetic fields. Sci. Adv..

[146] Takayama T., Hasegawa K., Izumi N., Kudo M., Shimada M., Yamanaka N., Inomata M., Kaneko S., Nakayama H., Kawaguchi Y. (2022). Surgery versus Radiofrequency Ablation for Small Hepatocellular Carcinoma: A Randomized Controlled Trial (SURF Trial). Liver Cancer.

[147] Simon C.J., Dupuy D.E., DiPetrillo T.A., Safran H.P., Grieco C.A., Ng T., Mayo-Smith W.W. (2007). Pulmonary radiofrequency ablation: Long-term safety and efficacy in 153 patients. Radiology.

[148] Cui R., Yu J., Kuang M., Duan F., Liang P. (2020). Microwave ablation versus other interventions for hepatocellular carcinoma: A systematic review and meta-analysis. J. Cancer Res. Ther..

[149] Liu X., Zhan Y., Wang H., Tang X., Cheng Y. (2025). Radiofrequency ablation versus microwave ablation for lung cancer/lung metastases: A meta-analysis. ANZ J. Surg..

[150] Issels R.D., Lindner L.H., Verweij J., Wessalowski R., Reichardt P., Wust P., Ghadjar P., Hohenberger P., Angele M., Salat C. (2018). Effect of Neoadjuvant Chemotherapy Plus Regional Hyperthermia on Long-term Outcomes Among Patients With Localized High-Risk Soft Tissue Sarcoma: The EORTC 62961-ESHO 95 Randomized Clinical Trial. JAMA Oncol..

[151] Overgaard J., Ccm Hulshof M., Dahl O., Arcangeli G. (2024). ESHO 1–85. Hyperthermia as an adjuvant to radiation therapy in the treatment of locally advanced breast carcinoma. A randomized multicenter study by the European Society for Hyperthermic Oncology. Radiother. Oncol..

[152] Dadfar S.M., Roemhild K., Drude N.I., von Stillfried S., Knüchel R., Kiessling F., Lammers T. (2019). Iron oxide nanoparticles: Diagnostic, therapeutic and theranostic applications. Adv. Drug Deliv. Rev..

[153] Mahmoudi K., Bouras A., Bozec D., Ivkov R., Hadjipanayis C. (2018). Magnetic hyperthermia therapy for the treatment of glioblastoma: A review of the therapy’s history, efficacy and application in humans. Int. J. Hyperth..

[154] Zhu M., Yang Z., Yu H., Zhu Q., Xu Y., Li Y., Li C., Zhao W., Liang Z., Chen L. (2020). The efficacy and safety of low-frequency rotating static magnetic field therapy combined with chemotherapy on advanced lung cancer patients: A randomized, double-blinded, controlled clinical trial. Int. J. Radiat. Biol..

[155] Liu Y.J., Liu X., Shu Y.S., Yu Y.G. (2024). Progress of the Impact of Terahertz Radiation on Ion Channel Kinetics in Neuronal Cells. Neurosci. Bull..

[156] Ren Z., Chen X., Cui G., Yin S., Chen L., Jiang J., Hu Z., Xie H., Zheng S., Zhou L. (2013). Nanosecond pulsed electric field inhibits cancer growth followed by alteration in expressions of NF-κB and Wnt/β-catenin signaling molecules. PLoS ONE.

[157] Perigo E.A., Hemery G., Sandre O., Ortega D., Garaio E., Plazaola F., Teran F.J. (2015). Fundamentals and advances in magnetic hyperthermia. Appl. Phys. Rev..

[158] Flores E.R., Sawyer W.G. (2024). Engineering cancer’s end: An interdisciplinary approach to confront the complexities of cancer. Cancer Cell.

[159] Korshoej A.R., Saturnino G.B., Rasmussen L.K., von Oettingen G., Sørensen J.C., Thielscher A. (2016). Enhancing Predicted Efficacy of Tumor Treating Fields Therapy of Glioblastoma Using Targeted Surgical Craniectomy: A Computer Modeling Study. PLoS ONE.

[160] Tammam E., Said A.M., Ibrahim A.A., Galal A.I.A. (2020). About the Interstitial Microwave Cancer Ablation: Principles, Advantages and Challenges. IEEE Access.

[161] Meric-Bernstam F., Mills G.B. (2012). Overcoming implementation challenges of personalized cancer therapy. Nat. Rev. Clin. Oncol..

[162] Korshoej A.R., Hansen F.L., Mikic N., von Oettingen G., Sørensen J.C.H., Thielscher A. (2018). Importance of electrode position for the distribution of tumor treating fields (TTFields) in a human brain. Identification of effective layouts through systematic analysis of array positions for multiple tumor locations. PLoS ONE.

[163] Rangan K., Briamonte C.B., Higgins M., Hoffman C., Holtzclaw S., McHugh M., Meyer A., Raber S., Schmus C., Seidl K. (2024). Neurocognitive side effects of pediatric brain tumors: A continuing challenge. Neuro-Oncology.

[164] Sun X.X., Yu Q. (2015). Intra-tumor heterogeneity of cancer cells and its implications for cancer treatment. Acta Pharmacol. Sin..

[165] Dakal T.C., Sharma N.K., Sharma A. (2024). Editorial: Revisiting the challenges and opportunities in cancer drug resistance. Front. Mol. Biosci..

[166] Ji X.M., Tian X.F., Feng S., Zhang L., Wang J.J., Guo R.W., Zhu Y.M., Yu X., Zhang Y.S., Du H.F. (2023). Intermittent F-actin Perturbations by Magnetic Fields Inhibit Breast Cancer Metastasis. Research.

[167] Li Z., Yu Q.J., Zhu Q.Y., Yang X.J., Li Z.B., Fu J. (2022). Applications of machine learning in tumor-associated macrophages. Front. Immunol..

[168] Besler E., Wang Y.C., Sahakian A.V. (2020). Real-Time Radiofrequency Ablation Lesion Depth Estimation Using Multi-frequency Impedance With a Deep Neural Network and Tree-Based Ensembles. IEEE Trans. Biomed. Eng..

[169] Briz P., López-Alonso B., Sarnago H., Burdío J.M., Lucía O. (2023). Tumor location on electroporation therapies by means of multi-electrode structures and machine learning. Bioelectrochemistry.

[170] Cui H., Zhao Y.Y., Wu Q., You Y., Lan Z., Zou K.L., Cheng G.W., Chen H., Han Y.H., Chen Y. (2024). Microwave-responsive gadolinium metal-organic frameworks nanosystem for MRI-guided cancer thermotherapy and synergistic immunotherapy. Bioact. Mater..

[171] Giladi M., Voloshin T., Shteingauz A., Munster M., Blat R., Porat Y., Schneiderman R.S., Cahal S., Itzhaki A., Kirson E. (2016). The antitumor activity of alternating electric fields (TTFields) in combination with immune checkpoint inhibitors. J. Clin. Oncol..

[172] Diamant G., Goldman H.S., Plotnitsky L.G., Roitman M., Shiloach T., Globerson-Levin A., Eshhar Z., Haim O., Pencovich N., Grossman R. (2021). T Cells Retain Pivotal Antitumoral Functions under Tumor-Treating Electric Fields. J. Immunol..

[173] Barsheshet Y., Voloshin T., Brant B., Cohen G., Avigdor L., Blatt R., Cahal S., Khalil T.H., Zemer-Tov E., Paz R. (2022). 860 *In vivo* effectiveness of tumor treating fields (TTFields) concomitant with immune checkpoint inhibitors in non-small cell lung cancer (NSCLC). J. Immunother. Cancer.

[174] Prasad R., Jain N.K., Conde J., Srivastava R. (2020). Localized nanotheranostics: Recent developments in cancer nanomedicine. Mater. Today Adv..

[175] Luiz M.T., Dutra J.A.P., Viegas J.S.R., de Araujo J.T.C., Tavares A.G., Chorilli M. (2023). Hybrid Magnetic Lipid-Based Nanoparticles for Cancer Therapy. Pharmaceutics.

[176] Singh B., Ma S.L., Hara T.O., Singh S. (2023). Nanomaterials-Based Biosensors for the Detection of Prostate Cancer Biomarkers: Recent Trends and Future Perspective. Adv. Mater. Technol..

[177] Kim Y., Chae J.K., Lee J.H., Choi E., Lee Y.K., Song J. (2021). Free manipulation system for nanorobot cluster based on complicated multi-coil electromagnetic actuator. Sci. Rep..

[178] Francisco A.C., del Mar S.A.M., Irene C., Sandra R.A., Josefa L., Elisa R.M., Nicolás O., Isabel N.M. (2013). Could Radiotherapy Effectiveness Be Enhanced by Electromagnetic Field Treatment?. Int. J. Mol. Sci..

[179] Dono A., Mitra S., Shah M., Takayasu T., Zhu J.J., Tandon N., Patel C.B., Esquenazi Y., Ballester L.Y. (2021). *PTEN* mutations predict benefit from tumor treating fields (TTFields) therapy in patients with recurrent glioblastoma. J. Neuro-Oncol..

[180] Pohling C., Nguyen H., Chang E., Schubert K.E., Nie Y., Bashkirov V., Yamamoto V., Zeng Y., Stupp R., Schulte R.W. (2023). Current status of the preclinical evaluation of alternating electric fields as a form of cancer therapy. Bioelectrochemistry.

[181] Sengupta S., Balla V.K. (2018). A review on the use of magnetic fields and ultrasound for non-invasive cancer treatment. J. Adv. Res..

